# Seroprevalence of IgG Rubella among Infants with Features Suggestive of Congenital Rubella Syndrome at a Tertiary Hospital in North Western Tanzania

**DOI:** 10.24248/eahrj.v6i1.680

**Published:** 2022-07

**Authors:** Elice C. Bendera, Adolfine Hokororo, Tumaini V. Mhada, Mariam Mirambo, Benson Kidenya, Dina C. Mahamba, Florentina Mashuda, Neema Kayange, Stephen E. Mshana

**Affiliations:** a Depatment of Paediatrics and Child Health-Bugando Medical Centre/Catholic University of Health and Allied Sciences, Mwanza Tanzania; b St Augustine Muheza Institute of Health and Allied Sciences; c Department of Microbiology and Immunology-Catholic University of Health and Allied Sciences, Mwanza Tanzania; d Department of Biochemistry - Catholic University of Health and Allied Sciences, Mwanza Tanzania; e Department of Paediatrics and Child Health, University of Dodoma College of Health Sciences

## Abstract

**Background:**

Congenital Rubella Syndrome (CRS) is among the causes of infant mortality and lifelong disability due to severe birth defects. There has been an increasing number of neonates born with congenital abnormalities suggesting CRS, at the same time the rubella seroprevalence among pregnant mothers and healthy school children in the northwestern Tanzania has been noted to be alarmingly high. This study aimed to determine prevalence of rubella antibodies and associated factors among infants suspected to have CRS.

**Methods:**

This cross-sectional study included 174 infants aged ≤ 12 months with at least one clinical features of CRS. The study was conducted between Septembers 2017 and March 2018 at Bugando Medical Centre, a consultant teaching hospital in North Western Tanzania. Collection of Social demographic and other relevant information was done hand in hand with screening for clinical symptoms suggestive of CRS and Blood samples were collected. Indirect enzyme-linked immunosorbent assay (ELISA) Test were conducted on collected sera to test for specific Rubella IgM and IgG antibodies.

**Results:**

The majority of enrolled infants were below 1 year of age; of these 83 (47.7%) were neonates and only 13.2% had received MR vaccine. Out of these, 111 (63.8%, 95%CI: 56.6-70.9) were IgG Rubella seropositive whereas none was IgM Rubella seropositive. In multivariate logistic regression analysis being neonate was the only factor that independently predicted rubella IgG seropositivity (OR 2.3; 95% CI 1.2 – 4.4; *p=0.012*).

**Conclusion:**

A significant proportion children (<12 months) with suspected CRS are IgG seropositive which is predicted by being a neonate (0-4weeks); this indicates high maternal seroprevalence and hence extended surveillance and measures to target women of child bearing age are recommended.

## BACKGROUND

Infection by Rubella virus usually causes a mild and self-limiting disease and about one half of individuals infected are asymptomatic, while symptomatic infections present with mild constitutional symptoms and rash.^1–3^ However, infection in pregnant women is of public health importance due to the teratogenic effects of rubella virus resulting into Congenital Rubella Syndrome (CRS).^2,4,5^

CRS refers to variable constellations of birth defects brought by intrauterine infection with the rubella virus.^1,2,5^ The risk of congenital infection and birth defects is high during the first 12 weeks of pregnancy.^6,7^ Once the virus crosses the placenta and access fetal circulation, it remains in the fetal blood stream for the remainder of the pregnancy and hence neonates born with CRS can secrete the virus after birth and can infect other infants and un-vaccinated adults.^8,9^

Features of CRS include cataracts, congenital heart disease, hearing impairment, pigmentory retinopathy and glaucoma. Others include microcephaly, purpura, hepatosplenomegally, meningoencephalitis, radiolucent bone diseases, jaundice within 24hours of birth and mental retardation.^1,10–12^

A suspected case of CRS is when an infant presents with any one or more of the mentioned symptoms whilethe probable case is when an infant presents withmore than one sign or symptom. The signs and symptoms in a probable case are grouped into major and minor categories, where presence of cataracts or congenital glaucoma, congenital heart disease (most commonly patent ductus arteriosus or peripheral pulmonary artery stenosis), hearing impairment, OR pigmentary retinopathy are considered as major while all the rest are minor categories. Presence of one major and any other minor sign without lab confirmation is considered as probable CRS.^6,9^ Also, presence of maculopapular rash, swollen lymphnode, arthralgia andconjunctivitis during pregnancy have been found to predict rubella infection in a mother.^9–11^

There has been increased admissions of neonates with congenital abnormalities suggesting CRS at Bugando Medical Centre (BMC),^13^ at the same time high seropositivity of Rubella seromarkers among pregnant women has been reported in this area.^14,15^ In addition about 11% of asymptomatic under five years children in the city of Mwanza were found to have acute rubella virus infection,^16^ this necessitates the need to investigate infants with features suggestive of CRS. Therefore, this study aimed at establishing the magnitude and the associated factors of Rubella IgM and IgG seroprevalence among infants born with features suggestive of CRS who are seen at the Bugando Medical Centre (BMC), which is a tertiary referral hospital in North Western Tanzania.

## METHODS

### Study Design and Study Site

This was a hospital based cross-sectional study conductedbetween September 2017 and March 2018 at BMC, a tertiary teaching and consultant hospital in North Western Tanzania. The study participants were from paediatric wards and paediatric outpatient clinic.

### Study Population

The study included all infants equal to or less than 12 months of age with at least one of the features suggesting CRS. The features included cataract(s), congenital glaucoma, congenital heart disease, pigmentory retinopathy, maculopapular rashes, hepatosplenomegaly, microcephaly, meningoencephalitis and jaundice that presented within 24 hours of birth. We excluded premature babies who had isolated Patent Ductus Arteriosus (PDA) because of high possibility of physiological occurrence.

Later on, during analysis we excluded children who missed rubella IgM, and IgG results due to insufficient blood sample.

### Sample Size Estimation

The minimum Sample size of 150 was calculated using the Leslie Kish formula for cross-sectional studies (17) as follows:


$$N=\frac{z^{2}p(1-p)}{D^{2}}$$

Where: Z = Score for 95% confidence interval = 1.96, P=Prevalence of 10.9% obtained from from Mirambo et al, D=Tolerable error = 0.05, and therefore N=0.109 (1-0.109) × (1.96 × 1.96)/(0.05 × 0.05)=148.8, however in order to attain the power to calculate for associated factors a total of 174 infants were recruited into this study.

### Screening Examination

All in and outpatient attendees aged less than or equal to 12 months were evaluated by the Principal Investigator and other research assistants who were medical doctors so as to look for any sign or symptom suggestive of CRS. Screening evaluation included patients' history and through physical examination this helped to reveal presence or even history of some of the specific clinical features of CRS. Simple fundoscopy using zyrev otoscope set (USA) was done to determine those children with congenital cataract, glaucoma and or pigmentory retinopathy. Occipital Frontal Circumference (OFC) was taken for all children to rule out microcephaly and echocardiogram to find out presence of congenital heart defects.

The OFC measurement was taken using a flexible tape measure by measuring over the most prominent part on the back of the head (occiput) and just above the eyebrows (supraorbital ridges).

Echocardiogram was done by the PI using GE 2D Echo Machine-INDIA to all enrolled infants to look for presence of congenital heart defects (CHD). All echocardiography images were crosschecked by an experienced paediatric cardiologist and those who were having structural normal findings continued with other management according to BMC protocol. Those who were found to have congenital heart disease were started on medical management and those who required heart surgery were referred to Jakaya Kikwete Cardiac Institute.

### Data Collection

Data was collected by the Principal Investigator with the help of the trained research assistant. Training of the research assistant and pretesting of the questionnaire were done prior to data collection, where under the observation of the PI the research assistant interviewed one parent using the questionnaire, unclear questions were corrected and the final pre tested questionnaire was formed. During actual data collection all children aged equal or less than 12 months were assessed, parents of those with one or more signs suggestive of CRS were approached and explanation of the study was given and were asked, if willing, to sign the consent. After receiving the written consent from the parentor guardian, information regarding residence, sex, age in weeks, date of birth of the enrolled infant was collected. Other information like age and relationship of a guardian, level of education, history of MR vaccination, marital status and clinical conditions was asked from the mothers and was also recorded. Presence of maculopapular rashes, hepatosplenomegally and jaundiceduring clinical assessment were evaluated and recorded. History of cataract, congenital heart disease, microcephaly, neonatal jaundice, meningoencephalitis and HIV status were also evaluated. Other information on clinical condition that we got from the mothers was any history of arthralgia, conjunctivitis, previous abortion, diabetes mellitus, prolong use of drugs, alcohol consumption, cigarette smoking and exposure to radiation or pollution.

### Laboratory Procedures

Blood samples were taken by using aseptic procedure whereby approximately 2cc of blood was collected into plain vacutainer tube. Serum was separated after centrifuging by using micropipette and kept in cryovials. Sera were separated, aliquoted into vials and stored at minus 40°C until processing. Detection of Rubella IgG and IgM antibodies was done by using indirect enzyme immunoassay (EIA) as per manufacturer's instructions (PISHTAZTEB DIAGNOSTICS, Iran Tehran). The PTRUBELLA IgM-ELISA has 100% sensitivity and 99% specificity whereas PT-RUBELLA IgG ELISA has 100% sensitivity and 100% specificity. The assay was done as per manufacturer's instructions. All individuals with rubella IgG concentration ≥10 IU/ml were considered as seropositive and those with concentrations below that threshold were considered seronegative for rubella while for IgM the cut-off index lower than 0.9 was considered as negative and those greater than 1.1 were considered as positive results.

### Data Management

Data were entered into a computer using Microsoft Excel 2010, cleaned and analysed using STATA version 13. Continuous variables were summarised using median (interquartile range). Logistic regression model was used to ascertain factors associated with IgG seropositivity. All factors with *P* value less than .20 on univariate analysis were subjected to multivariate logistic regression analysis. Crude (unadjusted) and adjusted odds ratios were calculated to quantify the strength of association between CRSseromakers and associated factors. At 95% confidence interval, *P* value of <.05 was considered as statistically significant.

### Ethical Consideration

The study was approved by the Joint CUHAS Bugando Ethical Committee with certificate no: CREC/231/2017. The aim and importance of the study was explained to parents/caretakers before recruitment was done. All parents/caretakerswho were willing to participate in the study signed informed consent. All information regarding the patients were kept confidential throughout the study. Patients’ records were kept such that their identities were not disclosed. All those who refused to participate in the study were given service and treatment regardless of their inclusion status in the study. The management of these patients was done according to the BMC Pediatric Department protocol on management of CRS.

## RESULTS

**FIGURE 1:**
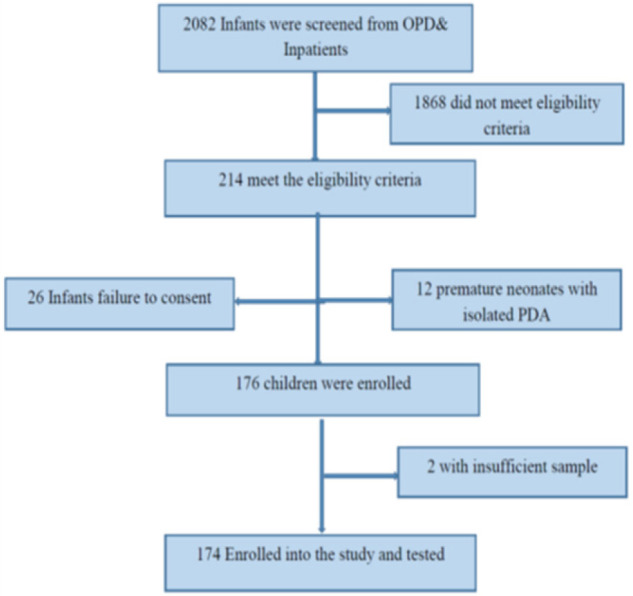
Recruitment Flow Chart

During the study period 2082 (1806 inpatients and 276 outpatients) infants attended and were screened for eligibility. A total of 214/2082 (10.3%) infants met the study eligibility criteriaiehad at least one clinical feature suggestive of CRS. Of these, 176/214 (82.2%) children were enrolled whereas 38 children were excluded for various reasons as summarised in the Figure 1, and among those eligible, 174 (81.5%) infants were included in the final analysis (Figure 1). The median age for the enrolled infants was 5.5 [1 – 24] weeks, and of the 174 analyzed only 23/174 (13.2%) received the Measles Rubella (MR) vaccination, 107/174 (61.5%) had CHD and of these 63/107 (58.9%) had PDA. There were 69/174 (39.7%) infants that had history of neonatal jaundice, while 42/174 (24.1%) had hepatosplenomegaly and 30/174 (17.2%) presented with maculopapular rashes. Moreover, there were 3/174 (1.7%) children with microcephaly, 3/174 (1.7%) with meningoencephalitis and 2/174 (1.1%) with cataracts. Table 1 illustrates additional demographic and clinical features of enrolled infants.

**TABLE 1:** Distribution of Socio Demographic and Clinical Characteristics of 174 Children with Features of Congenital Rubella Syndrome

Patients characteristics	Number (n)	Percent (%)
**Age of the child**		
Neonates (0-28 days)	83	47.7
Young Infants (1-3 months)	52	29.9
Infants (3-12 months)	39	22.4
**Sex of child**		
Female	94	54
Male	80	46
**Education of care giver**		
None/incomplete	25	14.4
Primary school	81	46.5
Secondary school	43	24.7
University/college	25	14.4
**Mothers age**		
18-28	96	55.2
29-39	66	37.9
40-50	12	6.9
**MR vaccine**		
No vaccination	151	86.8
**HIV Status**		
Negative	95	54.6
Positive	4	2.3
Unknown	75	43.1
**Clinical signs suggestive CRS**		
CHD	107	61.5
Jaundice	69	39.7
Rashes	30	17.2
Microcephaly	3	1.7
Cataract	3	1.7
Meningoencephalitis	2	1.1

The women whose infants were enrolledaged between 18-50 years with median age of 27 and IQR of [18 – 43]. Among them 29 (16.7%) had history of abortion, 8 (4.6%) had history of arthralgia and 8 (4.6%) had history of exposure to pollutants during pregnancy.

The women whose infants were enrolledaged between 18-50 years with median age of 27 and IQR of [18 – 43]. Among them 29 (16.7%) had history of abortion, 8 (4.6%) had history of arthralgia and 8 (4.6%) had history of exposure to pollutants during pregnancy.

### Prevalence of Rubella Seromarkers among Infants with Clinical Features Suggesting Congenital Rubella

Among the 174 infants investigated, 111/174 (63.8%) were IgG Rubella seropositive whereas none was IgM Rubella seropositive. Out of 174 infants investigated, 26/174 (14.9%) had clinical features suggesting probable CRS (had 2 or more features suggestive of CRS). When the PDA was considered as important CHD associated with CRS the probable CRS was 15/174 (8.6%).

### Factors associated with Rubella seropositivity among infants with features suggestive of CRS

On univariate analysis the factor associated significantly with Rubella seropositivity was being neonate (OR 2.3; 95% CI, 1.2 to 4.3; *P*=.012), whereas history of abortion in a mother had borderline significant association (OR 2.5; 95%CI, 0.9 to 6.5; *P*=.063. By multivariate logistic regression analysis, only being neonate independently predicted rubella IgG seropositivity (OR 2.3; 95% CI, 1.2 to 4.4; *P*=.012) as in Table 2.

**TABLE 2:** Factors Associated with Rubella IgG Seropositivity

Variable	Seropositive		Univariate		Multivariate	
	Yes (%)	No (%)	OR [95%CI]	Pvalue	OR [95%CI]	P value
**Gender**						
Female	59 (62.8)	35 (37.2)	1.0			
Male	52 (65.0)	28 (35.0)	1.1 [0.6–2.1]	0.76	1.1 [0.6–2.1]	0.739
**Age of child**						
Infants	50 (54.9)	41 (45.1)	1.0			
Neonates	61 (73.5)	22 (26.5)	2.3 [1.2–4 .3]	0.012	2.3 [1.2–4.4]	0.012^*^
**Hepatosplenomegally**						
No	88 (66.7)	44 (33.3)	1			
Yes	7 (87.5)	1 (12.5)	4.2 [ 0.5–34.7]	0.186	4.1[0.5 –36.6]	0.2
**History of Abortion**						
No	88 (60.7)	57 (39.3)	1.0			
Yes	23 (79.3)	6 (20.70	2.5 [0.9–6.5]	0.063	2.6 [1.0–6.8]	0.057

CI, confidence interval

## DISCUSSIONS

### Prevalence of Rubella Seromarkers and Probable CRS among Infants with Suspected CRS

These findings demonstrated a high seroprevalence of Rubella among infant population (63.8%) as a proxy of rubella seroprevalence among women of reproductive age.^6,10,11^ Placental transfer of maternal IgG antibodies to the foetus is an important mechanism that provides protection to the infant.^18^ The observed seroprevalence in the current study is higher than 32% and 34%, which were observed in Congo and Central African Republic, respectively.^19,20^ The high prevalence in our study might be explained by inclusion of neonates whereas these two studies included children above 6 months due to the fact that maternal IgG have been found to significantly disappear from 6 months of age.^6,21^ It should be noted that from neonates as the age increases, maternal IgG decrease by two fold in 4 weeks.^14,20,22^ The current seroprevalence is significantly higher than 1.8 observed in Kilimanjaro.^23^ The difference could be due to the assay used and population studied. The study in Kilimanjaro included asymptomatic children and they performed the ELISA from dried blood spots samples while the current study used serum. Previous studies have shown low sensitivity of dried blood spots samples in detecting Rubella specific IgG antibodies.^1^

Despite sufficient vaccination coverage in developed countries, high seroprevalence of rubella has been observed.^24^ The average prevalence of IgG rubella viruses was 70% in infants up to the age of six months, whereas it was around 95% in females of childbearing age. This prevalence was significantly high compared to our study for the reasons that mothers born after vaccination programme have been seropositive hence transfer the immunity to neonates.^24^

The majority 107 (61.5%) of our study participants had congenital heart disease and of these 63 (58.9%) had PDA. This correlates with findings from a cross sectional study done in Bangladesh where among assessed children 78% had congenital heart disease and among these 47.8% had PDA.^25^ The high prevalence could have been attributed to the fact that almost all these children attending to the particular institute were having cardiovascular malformations.^25^ Similarly, high prevalence of congenital heart disease in CRS has been observed also in a retrospective study which evaluated the common cardiovascular malformations in CRS.^26^

Our study demonstrated that the prevalence of probable CRS by using CHD as the indicator to be 14.6%. This is higher than one found in a retrospective study done in Philippines that identified probable cases 201 out of 4339 with estimated prevalence of 4.6%.^27^ The prevalence in this study was lower than ours due to the fact that being a retrospective study some clinical features might have been missed at the time of collection hence this led to the lower prevalence.

Our prevalence of probable CRS by using stringent criteria of using PDA was 8.6%. This was low compared to 20.6% found by Toizumi et al.^28^ The fact that Toizumi study was done during the time of an outbreak of rubella could have led to the higher prevalence compared to ours. However, this suggests the need of investigating for CRS in infants found to have CHD.

### Factors Associated with Rubella Seropositivity

By multivariate logistic regression analysis, only being neonate independently predicted rubella IgG seropositivity (OR 2.3; 95% CI, 1.2 to 4.4; P=.012) (Table 2). Transfer of maternal IgG antibodies to the foetus is an important mechanism that provides protection to the infant.^18^ Moreover, there is more evidence that placental IgG transfer depends on multiple factors such as maternal levels of total and specific IgG antibodies. Serological testing revealed that for neonates who tested positive for IgG antibodies to rubella, corresponds to the overall seropositivity rate of their mothers^29,30^. However these antibodies usually disappear in a period of 3-5months.^29^ This was also noted in the in Mwanza whereby pregnant women tested for rubella antibodies had the high seroprevalence of 92.6%.^14^ Similarly, in seroprevalenceassessment of rubella was reported to be 90.2% in Serbianneonates and this was found to predict the seroprevalence of rubella in their mothers.^30^

In conclusion, this study found that a significant proportion of neonates (63.8%) with suspected CRS are rubella IgG seropositive. In addition, a significant proportion of infants with suspected CRS can be grouped as probable CRS. Moreover, significantly high IgGseroprevalence in neonates indicates high maternal seroprevalence. Therefore, we recommend a continuous and sustained surveillance system on CRS on infants and children since the seroprevalence of CRS is still high. ThisCoordinated surveillance should involve other Units such as Ear Nose and Throat (ENT), Ophthalmology, Surgery andeven in maternity wards. In view of high proportion of neonates with rubellaseromarkers and CHD we recommend that any infant with suspected CRS should undergo cardiac evaluation by echocardiography. In addition to that these results call for revision in countries' vaccination policies, our results emphasize on the need to reconsider upper age limit for vaccination campaigns in developing countries where by screening and if possible vaccinating women in child bearing age may be cost-effective campaign to prevent CRS.
